# Fiber-Reinforcing Effect in the Mechanical and Road Performance of Cement-Emulsified Asphalt Mixtures

**DOI:** 10.3390/ma14112779

**Published:** 2021-05-24

**Authors:** Siyue Zhu, Zirui Xu, Xiantao Qin, Menghui Liao

**Affiliations:** 1 Department of Engineering Management, School of Civil Engineering and Architecture, Wuhan Polytechnic University, Wuhan 430023, China; 1613346608xzr@gmail.com (Z.X.); qinxiantao@whpu.edu.cn (X.Q.); 2 Department of Highway and Bridge Engineering, School of Transportation, Wuhan University of Technology, Wuhan 430063, China; wslmh1990@whut.edu.cn

**Keywords:** cement-emulsified asphalt mixtures, fiber-reinforcing effects, mechanical performance, road performance, microstructure

## Abstract

Cement-emulsified asphalt mixture (CEAM), a kind of cold mix asphalt mixture, has the advantages of energy conservation and emission reduction as well as easy construction. However, the performance of CEAM is not as good as hot mixed asphalt mixtures. Hence, in this study, two different fibers were adopted as the reinforcing phase to improve the comprehensive properties of CEAM. The results indicated that the addition proportion and curing time were crucial to fiber-reinforced cement-emulsified asphalt mixture (FRCEAM). The compressive strengths, water stability, and raveling resistances of FRCEAM preparations with polyester or brucite fibers (FRCEAM-PF and -BF, respectively) were enhanced significantly. FRCEAM-PF had the maximum flexural tensile strength and strain, which meant that its low-temperature performance was the best compared to FRCEAM-PF and CEAM. However, the contribution of PF to CEAM high-temperature stability was greater than that of BF. Fiber addition to CEAM not only enhanced the cycles of fatigue loading but also reduced sensitivity to changes in stress level. Furthermore, FRCEAM-BF durability was slightly better than that of FRCEAM-PF. SEM analysis indicated that fibers provided bridging and meshing effects. Although PF and BF showed different enhancement effects, both mixtures met the requirements for hot mixed asphalt mixtures.

## 1. Introduction

Cement-emulsified asphalt mixture (CEAM) has become a hot research topic under the subject of green transport because of its advantages in energy conservation and emission reduction, as well as easy construction [1,2,3]. Thus, much research has focused on improving the properties of cement-emulsified asphalt mortar and mixtures [4,5,6]. Specifically, mechanical properties, including indirect tensile strength (ITS), indirect tensile stiffness modulus, creep stiffness, and durability in term of water sensitivity of cement-emulsified asphalt mixture have been investigated [7,8,9,10]. Meanwhile, road performance, such as wheel-tracking and fatigue performance, have also been studied [11,12,13]. In addition, the microstructures of cement-emulsified asphalt binders or mixtures have been studied for enhancing adhesion between aggregate and mastic [14,15]. However, the properties of CEAM are worse than those of hot mixed asphalt mixtures, which limits the former’s wide application for high-grade highways. Specifically, Zarei et al. [1] have indicated that low-temperature performances of semi-flexible pavement (SFP) is still an issue in application. Wang et al. [16] have proposed and tried to resolve adhesion decline and road performance decreases of cement-emulsified asphalt mixture in the process of application. Xu et al. [17] have suggested that the fatigue resistance and anti-cracking resistance of cold-mixed mixtures should be further improved. Thus, here, modified emulsified asphalt was examined, with fiber additives adopted as the reinforcing phase, and mechanical and road performance of fiber reinforcement on cement-emulsified asphalt mixtures was comparatively examined.

## 2. Materials and Methods

### 2.1. Materials and Gradation

#### 2.1.1. Materials

P.R.7.5 Cement (Jidong Cement Co., Ltd., Tangshan, China) was selected for this study. The basic technical parameters, following the standard “Road Portland cement” (GB/T-13693-2017) [18], are shown in Table 1. The technical indicators of polyester (PF) and brucite fibers (BF) used in the present tests are shown in Table 2, following the Chinese specification “fiber for asphalt pavements” (JT/T 533-2020) [19]. A self-developing, slow breaking, and quick-setting styrene–butadiene–styrene (SBS) modified emulsified asphalt was used here. Then, the technical indicators were evaluated, following standard “Technical Specification for Construction of Highway Asphalt Pavements” (JTG F40-2017) [20], with the results listed in Table 3. The properties of aggregates and mineral powder, which meet the requirements of JTG F40-2017, are indicated in Table 4 and Table 5.

#### 2.1.2. Gradation

Compared with other hot mixed asphalt mixtures used in road surfaces, the cohesive force of cementitious materials in CEAM require a relatively long development process. In addition, the strength of these mixtures increases with aging. Xiao has proposed that the dense gradation used in CEAM has better adhesion, and the strength of fully emulsified asphalt and cement hydration degree were high [21]. For these reasons, 13 mm nominal maximum size of aggregate, dense graded asphalt concrete (AC-13) was adopted in this test, with the gradation shown in Table 6.

### 2.2. Preparation of the Specimens

In this study, experiments examined fiber-reinforcing effects in CEAM mechanical and road performance. Polyester and brucite fibers (PF and BF, respectively) were chosen as the reinforcing phase to prepare fiber-reinforced cement emulsified asphalt mixtures (FRCEAM), named as FRCEAM-PF and -BF, respectively. Through a previous study, the optimal amounts of BF and PF in FRCEAM were determined to be 0.2 and 0.3 wt % of aggregate, respectively [22], and CEAM was considered as a reference. Three mixtures of a 5.0 wt % asphalt/aggregate ratio were used in experiments, and the mass ratio of cement to residual asphalt (C/A) in emulsions was 1/1. Two compaction methods were used for each mixture, one is by the Marshall compaction method, for the preparation of samples for the Marshall stability (MS), indirect tensile, compressive, water stability, and Cantabro tests. The other was a wheel compaction method for preparing samples for low-temperature bending, rutting, and beam flexural fatigue tests. Different specimens of CEAM and FRCEAM were made as follows. First, aggregates and fiber (if used) were added in an asphalt mixer and mixed for 30 s Next, 2 wt % water (per aggregates) was gradually added and mixed for 30 s Next, self-developing SBS-emulsified asphalt was added and mixed for 3 min, and cement was added and mixed for 30 s. For the Marshall compaction method, the mixture was compacted with a Marshall compaction hammer with 35 blows to each side. The product was held at room temperature (RT, 23 ± 2 °C) for 24 h and compacted again, with 40 blows to each side, and then demolded. For the wheel compaction method, the mixture was compacted with a rolling wheel with 6 round trips (12 times), following the Chinese standard “Standard test methods of Bitumen and Bituminous Mixture for Highway Engineering” (JTG E20-2011) [23]. Then, the product was held at RT for 24 h and compacted again with a rolling wheel with 6 round trips and then demolded. Finally, samples were cured at RT for 7 and 28 d.

Different specimens of CEAM and FRCEAM used for SEM observations were prepared as follows: first, emulsified asphalt, cement, and fiber were weighed in proportion. Then, cement was added slowly into the emulsified asphalt while stirring slowly. Then, the mixture was stirred at high speed for 3 min, after which the fiber (if included) was added into the mixture at low speed and then stirred at high speed for 3 min. Next, the mixture was poured into a silicone mold for forming. Finally, samples were cured at RT for 28 d.

### 2.3. Test Methods

#### 2.3.1. Mechanical Performance Tests

Examination of fiber-reinforcing effects on CEAM mechanical properties were carried out through Marshall stability (MS), ITS, and compressive tests. Before tests, Marshall specimens of CEAM, FRCEAM-BF, and FRCEAM-PF were prepared after curing for 7 and 28 d. MS and ITS tests were conducted according to Chinese specification JTG E20-2011. Compressive tests were performed on a Universal Testing Machine (SANS series, MTS Co., Ltd., Shenzhen, China) to obtain the complete compressive stress–strain (CSS) curves of an emulsified asphalt mixture (EAM), CEAM, and FRCEAMs at 7 d. Tests were carried out at 20 °C and the stress control mode was chosen with a loading rate of 2 mm/min. Compressive strengths of the FRCEAM samples were calculated using data from the resulting loading–deformation curves.

#### 2.3.2. Road Performance Tests

Marshall immersion tests were used to measure the water stability of FRCEAM following the Chinese specification JTG E20-2011. In these experiments, Marshall specimens of CEAM, FRCEAM-BF, and FRCEAM-PF at 7 and 28 d of age were prepared and each was divided into two groups of samples. Then, the standard Marshall stability at 60 °C for 30 min and 48 h immersion (MS and MS_1_, respectively) were measured. The immersion residual Marshall stability (MS_0_) was used to evaluate FRCEAM water stability.

FRCEAM is a cold mixed asphalt mixture, with adhesion between the binder and aggregate usually inferior to that of hot asphalt mixtures, adhesion thus being an important aspect of FRCEAM durability. To analyze the adhesion property of FRCEAM, Cantabro raveling tests were used. Before tests, Marshall specimens of CEAM, FRCEAM-BF, and FRCEAM-PF at 7 and 28 d were prepared and held in a constant temperature water bath of 2 ± 0.5 °C for 20 h. Then, the specimens were mounted in a Los Angeles test machine (DCS Testing & Equipment Co., Wilmington, DE, USA) at a speed of 30–33 rpm for 300 rotary hits. The raveling loss of each mixture was calculated according to Chinese specification JTG E20-2011.

The low-temperature performance of FRCEAM was assessed in low-temperature bending tests according to Chinese standard JTG E20-2011. Before tests, each specimen of CEAM, FRCEAM-BF, and FRCEAM-PF at 7 and 28 d was prepared with dimensions of 250 ± 2.0 mm (l), 30 ± 2.0 mm (w), and 35 ± 2.0 mm (h). Testing was carried out at −10 °C with the loading rate at 1 mm/min. The flexural tensile strength (R_B_), maximum flexural tensile strain (ε_B_), and flexural stiffness modulus (S_B_) were used to evaluate FRCEAM low-temperature performance. 

The influence of fiber on the high-temperature performance of FRCEAM mixtures was evaluated using rutting tests. Before experiments, specimens of CEAM, FRCEAM-BF, and FRCEAM-PF at 7 and 28 d were prepared with dimensions of 300 mm × 300 mm × 50 mm. The tests were performed at 60 °C, and the dynamic stabilities of each mixture were calculated according to Chinese specification JTG E20-2011.

Beam flexural fatigue tests were used to test FRCEAM fatigue performance. Taking into account the sensitivity of FRCEAM performance to aging, all specimens of CEAM, FRCEAM-BF, and FRCEAM-PF were formed by the wheel-rolling method (JTG E20-2011), cured for 28 d, and then samples cut into 250 mm × 40 mm × 40 mm beams. Flexural fatigue tests were carried out on a Universal Testing Machine 100 kN cap (UTM-100), at ambient temperature (15 °C), stress control mode chosen, and loading frequency at 10 Hz. The fatigue life of the mixture was related to the initial tensile stress, as in Formula (1).

(1) $$N_{f}=K{\left(\frac{1}{\sigma_{0}}\right)}^{n}$$

where *N_f_* is the fatigue life, *σ*_0_ is the initial flexural tensile stress (MPa), *K* and *n* are the regression coefficients.

From Formula (1), the fatigue life of the mixture and initial flexural tensile stress satisfy the linear relation in double logarithmic coordinates, as shown in Formula (2).

(2) $${\ln N}_{f}=\ln K\;-\;n\mathrm{nln}\sigma_{0}$$

#### 2.3.3. Microstructurual Tests

For microstructural observations, SEM was performed using an S-4800 (High-Technologies Corp., Tokyo, Japan). Before examinations, the samples were cut into 5 mm × 5 mm sections and sputtered with 10 nm of gold.

## 3. Results and Discussion

### 3.1. Fiber-Reinforcing Effects on CEAM Mechanical Properties

#### 3.1.1. MS of FRCEAM

**Figure 1:**
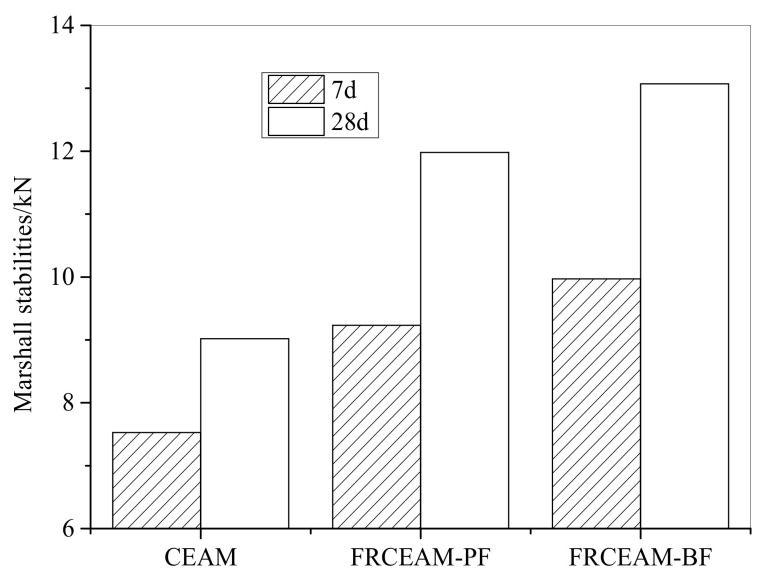
MS of CEAM and FRCEAM.

The MS of CEAM and FRCEAM showed that compared with CEAM, the MS of FRCEAM-PF and -BF increased by 22.6 and 32.4% at 7 d and increased by 32.8 and 44.9% at 28 d, respectively (Figure 1). The fiber-reinforcing effects on the MS of CEAM were clearly increased and the BF effects improved the CEAM MS higher than that of PF. When the curing age increased from 7 to 28 d, the MS of CEAM, FRCEAM-PF, and FRCEAM-BF increased by 19.8, 29.8, and 31.1%, respectively. This showed that the increased range of MS of the two modified FRCEAM samples was significantly greater than that of CEAM with increased curing.

#### 3.1.2. ITS of FRCEAM

**Figure 2:**
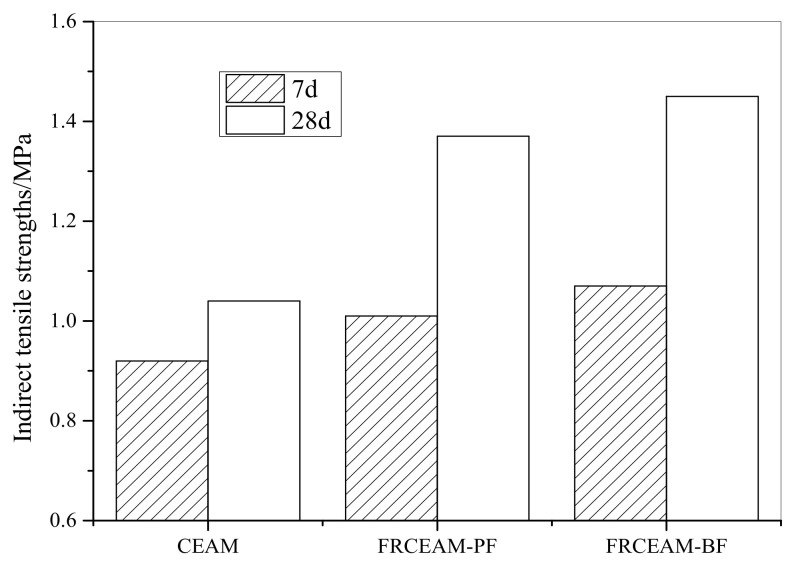
ITS of CEAM and FRCEAM.

Evaluation of the ITS of CEAM and FRCEAM showed that the ITS of FRCEAM-PF and -BF were slightly increased by 9.8 and 16.3% at 7 d and increased by 31.7 and 39.4% at 28 d, respectively, compared with that of CEAM (Figure 2). When the curing age increased to 28 d, CEAM ITS only increased by 13%, while that of FRCEAM-PF and -BF increased by 35.6 and 35.5% respectively, which were much higher than that of CEAM. Visibly, the curing age was crucial to ITS growth of FRCEAM. With increased age, fiber and cement hydration products, fiber and asphalt, and fibers in FRCEAM combined with each other to form a three-dimensional (3D) interpenetrating network structure, which greatly improved the mixture ITS.

#### 3.1.3. Complete CSS Curves of FRCEAM

**Figure 3:**
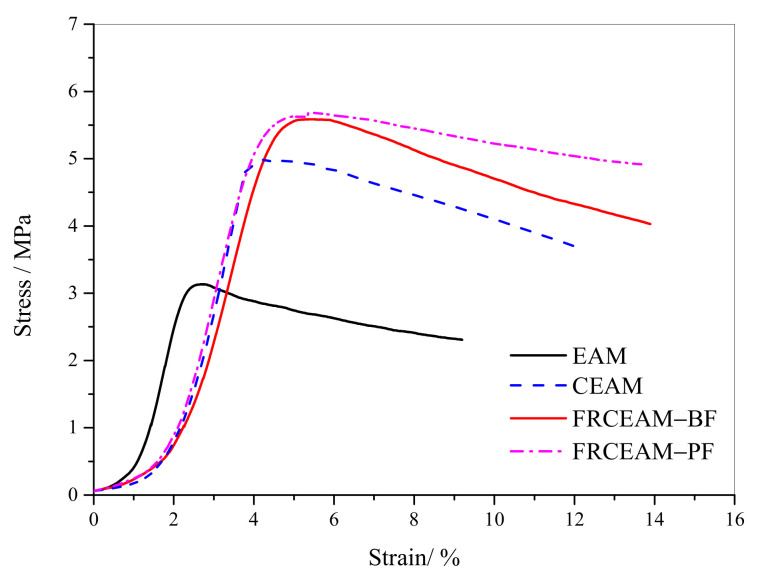
Complete CSS curves of CEAM and FRCEAM.

To intuitively express the typical mechanical characteristics of FRCEAM, the CSS curves of EAM (C/A = 0), CEAM, FRCEAM-PF, and FRCEAM-BF were examined. The CSS curves of EAM were observed to possess very similar geometric characteristics to a typical CSS curve of asphalt concrete (Figure 3). Under initial stress, the CSS curve of EAM also had a clear reverse bending section, and then, the stress entered a period of rapid growth, with the stress–strain relationship becoming linear, after which the CSS entered the hyperbolic segment, eventually reaching a maximum. Compared with EAM, the CSS curves of CEAM and the two FRCEAMs were similar in geometry, but there were clear differences in many details.

First, compared with EAM, the reverse bending section of CSS curves of CEAM and FRCEAM were relatively wide. This meant that the strain development speed was faster than that of EAM in the initial stage of stress. This was because the addition of fiber and cement introduced more voids to the mixture, especially because the effects of cement hydration on mixture void content were more apparent. Hence, the strain of FRCEAM increased faster at lower stress levels in the initial stage of load action.

Second, the highest points of the CSS curves of CEAM and FRCEAM were significantly higher than that of EAM, and the highest point of the curves for CEAM was lower than that of FRCEAM. The compressive strength of FRCEAM was significantly higher than that of EAM as well as larger than CEAM, because with the contribution to cement hydration products, asphalt formed better bonding after demulsification and fiber composite system creation, which added to the mixture compressive strength, which were significantly increased compared with pure emulsified asphalt material. Meanwhile, fiber addition thus made the peak strain increase.

### 3.2. Fiber-Reinforcing Effects on CEAM Road Performance

#### 3.2.1. Water Stability of FRCEAM

**Figure 4:**
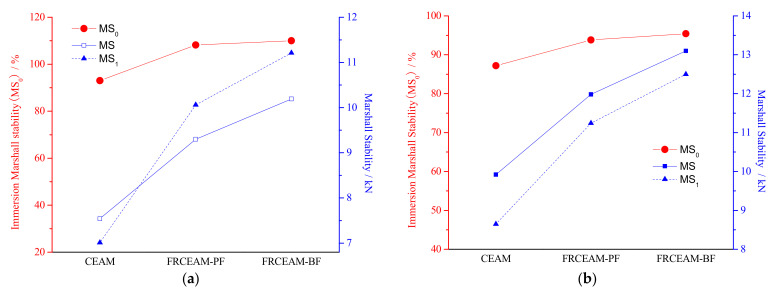
Marshall immersion tests results of CEAM and FRCEAM. (**a**) 7 d; (**b**) 28 d.

The results of Marshall immersion tests for FRCEAM at 7d (Figure 4a) showed that the MS and MS_1_ of the two FRCEAMs were higher than those of CEAM. After a 48 h immersion, the MS_1_ of FRCEAM-PF and -BF were even greater than its MS. That is to say, the MS_0_ of FRCEAM-PF and -BF at 7 d was more than 100% and reached 108.2 and 110.0%, respectively. The reason for this phenomenon was that the specimen strength had not been fully formed at 7 d and thus, the increase rate of MS of FRCEAM was greater than the adverse effects of water erosion on its MS at 60 °C at 48 h, which led to the MS_0_ of FRCEAM being greater than 100%. For CEAM, although its strength increased with age, without fiber reinforcement, the MS_0_ was only 93.0%. This comparison showed that fiber addition was very beneficial for improving CEAM water stability.

The MS of FRCEAM was still significantly higher than that of CEAM, which was similar to the test results at 7 d (Figure 4b). However, the difference here was that the MS of all mixtures decreased after 48 h of immersion, with the MS_0_ of CEAM, FRCEAM-PF, and -BF at 87.2, 93.8, and 95.4%, respectively. Compared with CEAM, fiber addition in CEAM resulted in a 3D network structure, between the cement and emulsified asphalt, that was denser and thus improved CEAM water stability. The effects of PF and BF on the immersion residual stability of the mixtures were small.

#### 3.2.2. Cantabro Raveling Loss Resistance of FRCEAM

**Figure 5:**
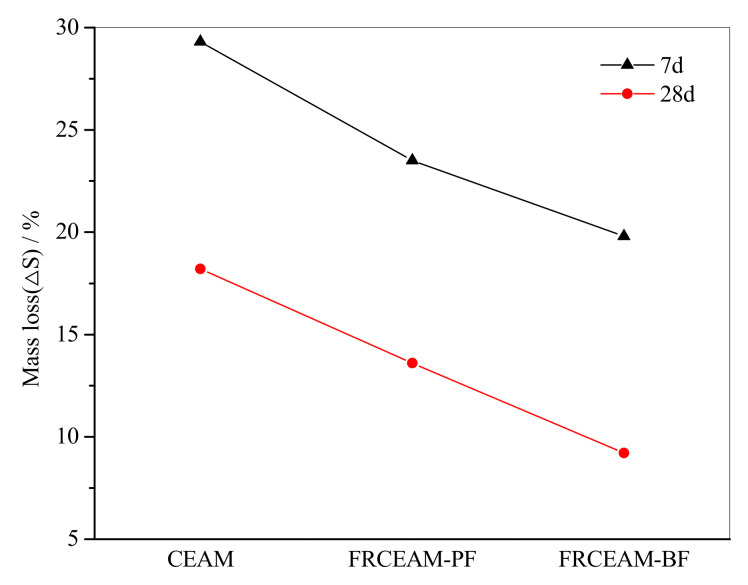
Cantabro raveling losses of CEAM and FRCEAM.

Examination of Cantabro raveling losses of FRCEAM showed that at 7 d, the raveling losses of the two FRCEAMs were much smaller than that of CEAM, with the raveling losses of CEAM, FRCEAM-PF, and -BF at 29.3, 23.5, and 19.8%, respectively (Figure 5). At 28 d, the mass losses of the three mixtures were <20%, with the raveling losses of CEAM, FRCEAM-PF, and -BF at 18.2, 13.6, and 9.2%, respectively. Adhesion between aggregate and cementing material in CEAM increased significantly with increased curing age. In particular, adhesive force was further enhanced by fiber addition, which reduced the possibility of spalling, and the raveling loss of the mixtures fell to a lower level. At 28 d, the mass loss of FRCEAM specimens met the requirements of hot blended asphalt mixture specifications, which showed that fiber-reinforced cement emulsified asphalt composite materials could play a good role in aggregate adhesion.

#### 3.2.3. Low-Temperature Performance of FRCEAM

**Figure 6:**
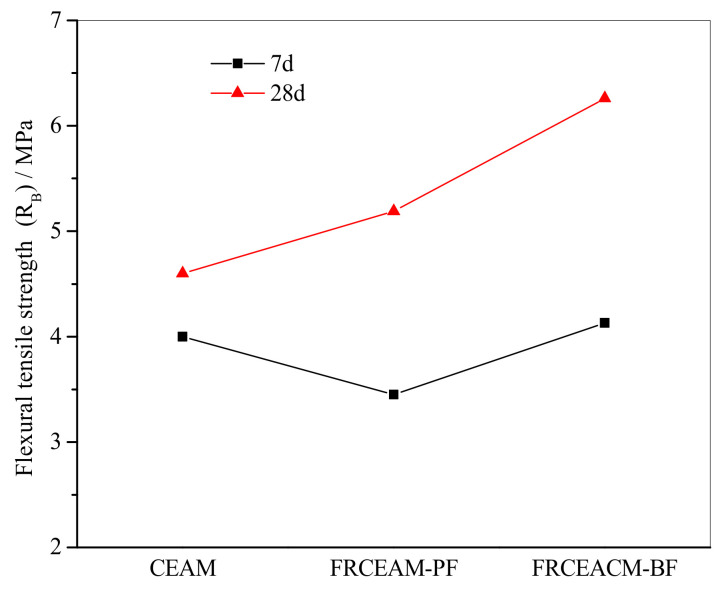
Flexural tensile strengths of CEAM and FRCEAM.

Examination of the R_B_ of FRCEAM at 7 and 28 d showed that at 7 d, the difference between the R_B_ of the three mixtures were small, while the R_B_ of FRCEAM-BF was the largest, CEAM was medium, and FRCEAM-PF was the smallest (Figure 6). After curing for 28 d, the R_B_ of the three mixtures increased significantly, with the increase for CEAM, FRCEAM-PF, and -BF at 15.0%, 50.4%, and 51.6%, respectively. This reflected that in a short period of time, the fiber reinforcement effect in CEAM was not effectively reflected, such that the R_B_ of FRCEAM-PF at 7 d was even smaller than that of CEAM. However, as the strength of emulsified asphalt and cement increased with age, the anchoring force between fiber and components in the composite system was gradually enhanced and adhesion between the aggregate and binder was further enhanced. Thus, the R_B_ values of these FRCEAMs were greatly improved.

The ε_B_ values of the three mixtures were large and significantly larger than the requirements of ε_B_ at low temperature for hot blended asphalt mixture (JTG F40-2017, Figure 7). In combination with the R_B_ and ε_B_ of FRCEAM, the load level of CEAM and FRCEAM was lower than that of hot blended asphalt mixture during loading. However, the deflection of CEAM and FRCEAM increased continuously in a larger range without fracture at lower load levels, such that the ε_B_ of the mixtures were larger. In addition, the ε_B_ of FRCEAM-PF at 7 and 28 d were smaller than that of CEAM and the ε_B_ of FRCEAM-BF was the highest of the three mixtures.

**Figure 7:**
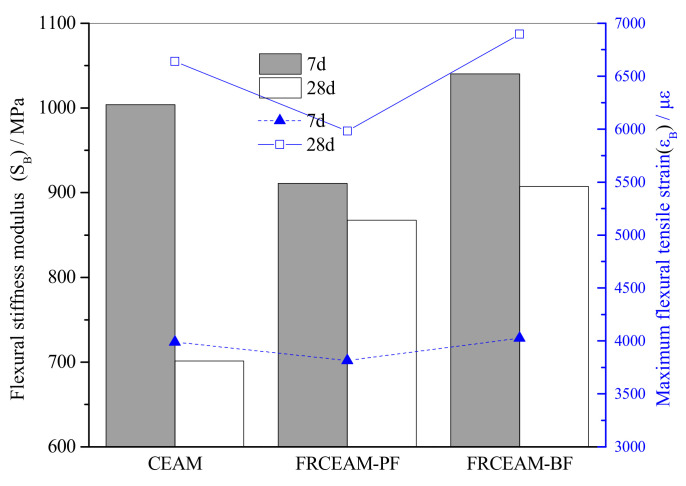
Maximum flexural tensile strains and flexural tensile stiffness modulus of CEAM and FRCEAM.

The S_B_ of FRCEAM at 7 and 28 d showed that when curing increased from 7 to 28 d, the R_B_ and ε_B_ of the three mixtures increased. Meanwhile, the S_B_ decreased to a certain extent (Figure 6 and Figure 7). Thus, the low-temperature crack resistance performance of the mixtures clearly improved with age.

#### 3.2.4. High-Temperature Performance of FRCEAM

**Figure 8:**
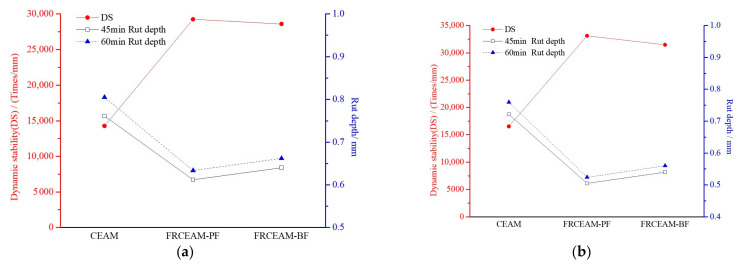
Dynamic stabilities of FRCEAM. (**a**) 7 d; (**b**) 28 d.

Examination of rutting test results for CEAM and FRCEAMs at 60 °C at 7 and 28 d showed that at 7 d, the rutting depth of CEAM and FRCEAMs with two kinds of fiber at 45 and 60 min were <1 mm, and the dynamic stability values of CEAM, FRCEAM-PF, and -BF at 14,268, 29,250, and 28,584 mm^−1^, respectively (Figure 8a). Such high dynamic stability was comparable to hot mix asphalt mixtures using gap-graded or high viscosity modified asphalt. When curing reached 28 d, the deformation of the three mixtures was further reduced, and their dynamic stability further increased, reaching 16,538, 33,116, and 31,460 mm^−1^, respectively (Figure 8b). Combined with rutting test results of the mixtures at two different ages, fiber addition increased high-temperature stability more than one-fold, and the contribution of PF to CEAM high-temperature stability was greater than that of BF. In general, the high-temperature stability of the three mixtures reached a high level compared with ordinary hot mix asphalt mixture AC-13, indicating that as a cementitious material, cement-emulsified asphalt composites, especially with added fibers, even a degradable fiber type that was not conducive to rutting resistance, exhibited excellent high-temperature stability.

#### 3.2.5. Fatigue Performance of FRCEAM

**Figure 9:**
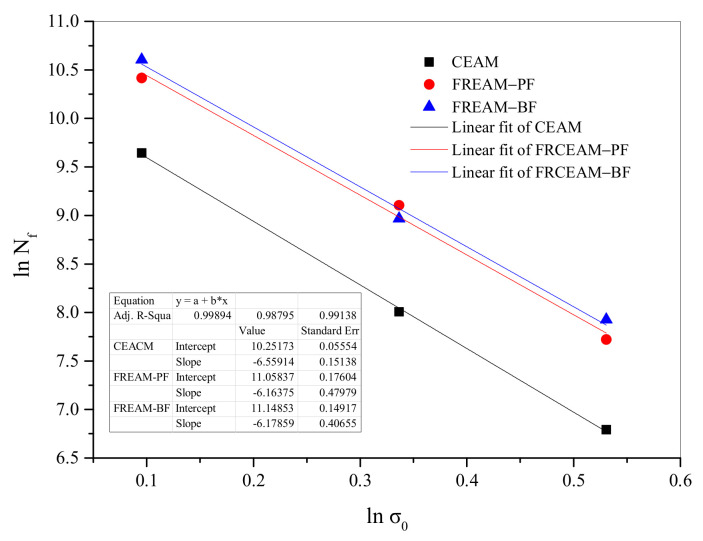
Comparison for fatigue performance of CEAM and FRCEAM.

The results of fatigue test regression analysis for CEAM and FRCEAM showed that the regression coefficient *K* in Formulas (1) and (2) indicated the height of the fatigue curve (Figure 9). The greater the *K* value, the better the fatigue performance. The *n* value represented the fatigue curve slope, and the larger the *n* value, the higher the sensitivity of the mixture fatigue life to changes in stress level and, thus, the worse the fatigue performance. From parameter analysis of the regression equation, all correlation coefficients (*R*^2^) of the three mixture were >0.98, which indicated that the number of fatigue failure cycles at three stress levels were linearly correlated with the initial stress in the double logarithmic coordinates.

The order of *K* from large to small was FRCEAM-BF, -PF, and CEAM, which showed that fiber addition to CEAM improved mixture fatigue performance. Then, from the regression coefficient *n*, CEAM was the largest (*n* = 6.559), which indicated that the sensitivity of the mixture to changes of the corresponding force was greater. The *n* values of FRCEAM-PF and -BF were 6.164 and 6.179, respectively, which illustrated that fiber addition in CEAM not only enhanced the tolerated cycles of fatigue loading but also reduced the sensitivity to changes in stress level.

In the process of fatigue testing, the applied external load causes stress concentration inside a mixture and, when the cycles of load accumulate to a certain stage, microcracks gradually occur inside the mixture. Furthermore, the continuous development of microcracks slowly extends to the mixture surface and gradually run throughout, resulting in structural failure. After adding fiber to CEAM, a large number of fibers were distributed in the mixtures. Taking FRCEAM-PF as an example, the large number of fibers distributed in the mixture was clearly seen at the failure interface after a fatigue test, with the fibers effectively delaying crack generation and preventing crack expansion. As a result, the fatigue load resistance performance of the two FRCEAMs was improved to varying degrees. Thus, comparing the fatigue equation regression coefficient of FRCEAM with two different fibers, the durability of FRCEAM-BF was seen to be slightly better than that of FRCEAM-PF.

Finally, when stress exceeded the compressive strength and entered the descending part of the curve, the stress of FRCEAM-BF was observed to decrease faster than that of FRCEAM-PF. This could be considered to be determined by the characteristics of the fiber itself. The ratio of length to diameter of PF was larger, and the delay failure duration in the failure stage of the concrete specimen was longer compared to BF. Therefore, the decreasing section of the stress–strain curve of FRCEAM-PF was gentler than that of the other two mixtures.

### 3.3. The Microstructure of FRCEAM

**Figure 10:**
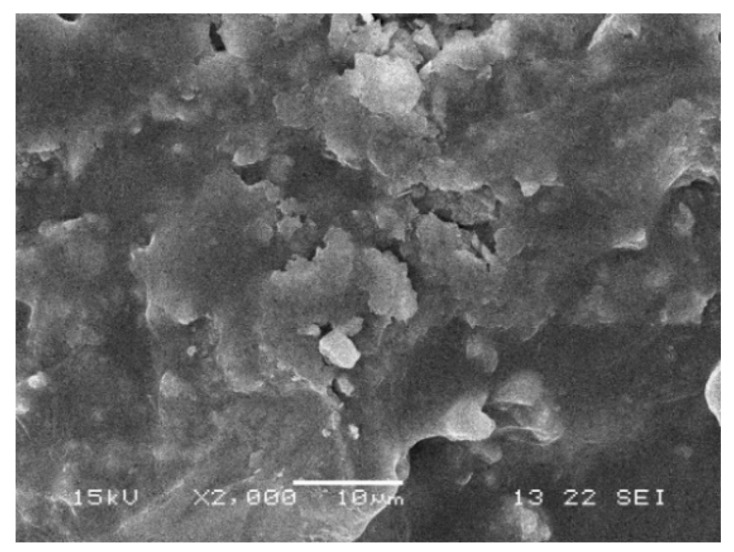
Microstructure of CEAM.

**Figure 11:**
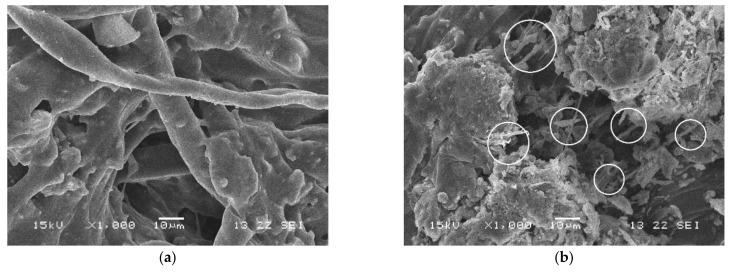
The microstructure of FRCEAM. (**a**) FRCEAM-PF; (**b**) FRCEAM-MF.

CEAM was seen to form a network structure of mutual penetration and multi-point contact in the composite system, which was beneficial to the composite effect of the material (Figure 10). The polyester fibers were observed to overlap with each other, the cement hydration products are inserted at both ends, and the fibers in asphalt are wound (Figure 11a). This fiber reinforcement effect had a positive effect on the mechanical properties of CEAM. Due to the large range of length and diameter of brucite fibers, the number of brucite fibers with different lengths and diameters were more and distributed in the composite system, yielding the bridging and meshing effects more prominently (Figure 11b).

## 4. Conclusions

This study investigated the fiber-reinforcing effects of added fibers in the mechanical and road performance of CEAM by a series of laboratory tests including MS, ITS, compressive, water stability, SEM, Cantabro, low-temperature bending, rutting, and beam flexural fatigue tests. The performance of these mixtures after curing was evaluated in terms of raveling resistance, moisture susceptibility, rutting resistance, low-temperature crack resistance, and fatigue resistance. Based on the experimental results, the following conclusion were drawn:

1. Fiber addition effectively enhanced the mechanical properties of CEAM, with the 28 d of curing MS of FRCEAM-PF and -BF increased by 32.8% and 44.9% and ITS increased by 31.7% and 39.4%, respectively. Curing was crucial to the mechanical property growth of these FRCEAMs. When the curing age increased from 7 to 28 d, the MS of FRCEAM-PF and -BF increased by 29.8% and 31.1% and ITS increased by 35.6% and 35.5%, respectively.
2. The compressive strengths of FRCEAM-BF and FRCEAM-PF were greater than that of CEAM and significantly higher than EAM. Meanwhile, fiber addition made the peak strain increase. When the stress exceeded the compressive strength and entered the descending section, the stress of FRCEAM-BF decreased faster than that of FRCEAM-PF.
3. Fiber addition enhanced CEAM water stability, with the MS_0_ of CEAM, FRCEAM-PF, and -BF at 87.2%, 93.8%, and 95.4%, respectively. In addition, PF and BF exhibited little difference in their effects on residual stability.
4. At 7 d, the raveling loss of CEAM was 29.3%, with the raveling loss of FRCEAM-PF and -BF at 23.5% and 19.8%, respectively. At 28 d, the raveling losses of CEAM, FRCEAM-PF, and -BF were all < 20% and reaching 18.2%, 13.6%, and 9.2%, respectively, which met the requirements for hot blended asphalt mixtures.
5. The ε_B_ of CEAM, FRCEAM-PF, and -BF were large and significantly larger than the requirements for hot blended asphalt mixtures (JTG F40-2017). The ε_B_ of FRCEAM-PF at 7 and 28 d were smaller than that of CEAM, but the ε_B_ of FRCEAM-BF was the highest of the three mixtures. When the curing time increased from 7 to 28 d, although the R_B_ and ε_B_ of the three mixtures increased, their S_B_ decreased to a certain extent. Thus, the low-temperature crack resistance performance of these mixtures clearly improved with age.
6. The high-temperature performance of CEAM, FRCEAM-BF, and -PF was excellent at 7 d. At 28 d, deformation of the three mixtures was further reduced, and their dynamic stability also increased. Fiber addition increased the high-temperature stability more than one-fold, with the dynamic stability of FRCEAM-PF and -BF reaching 33,116 and 31,460 mm^−1^, respectively, at 28 d. The contribution of PF to CEAM high-temperature stability was greater than that of BF.
7. Fiber addition to CEAM not only enhanced the tolerated cycles of the fatigue loading but also reduced sensitivity to changes in stress level. Furthermore, the durability of FRCEAM-BF was slightly better than that of FRCEAM-PF.

From this study, the bridging and meshing effect of PF and BF fibers addition were clearly seen to greatly improve the mechanical and road performance of CEAM, which could promote the application of cold mix asphalt mixtures, and it provides an idea for fiber selection in CEAM. In future studies, more types of fibers need to be examined to expand this concept so as to further improve the overall properties of CEAM.

## Figures and Tables

**Table 1:** Basic technical indicators of cement.

Indicators	Test Values	Technical Requirements
Specific surface area (m^2^·kg^−1^)	390	300–450
Initial setting time (min)	130	≥90
Final setting time (min)	600	≤720
Dry shrinkage rate (%)	0.04	≤0.1
28 d abrasion value (m^2^·kg^−1^)	1.5	≤3.0
3 d compressive strength (MPa)	26	≥21
28 d compressive strength (MPa)	49.0	≥42.5
3 d Bending Strength (MPa)	4.7	≥4
3 d Bending Strength (MPa)	10.7	≥7.5

**Table 2:** Basic technical characteristics and requirements of the two fibers.

	Fiber Type	Polyester Fiber	Brucite Fiber	Technical Requirements
Items				
Color and appearance		White, Sarcinform	Hoary, Cottony	-
Average length (mm)		6	0.2–4	10–38/≤6
Average diameter (μm)		20	2–4	15–35/≤5
Density(g/cm^3^)		1.316	2.284	-
Moisture content (%)		0.05	0.08	≤0.2
Oil absorption rate (times)		4.2	3.6	≥0.5
Breaking strength (MPa)		360	-	≥270
Elongation at break (%)		12	-	≥8
Crimp fiber content (%)		1	-	≤3
Shot content (0.15 mm) (%)		-	8	≤20
Passing rate of 0.15 mm sieve (%)		-	65	60 ± 10
Added value of the passing rate of 0.15 mm sieve (%)		-	18	≤22

**Table 3:** Basic technical indexes of SBS-modified emulsified asphalt.

Indicators	Test Values	Technical Requirements
80 μm sieving residue (%)	0.02	≤0.2
Engrass viscosity E25	15	3–30
Content of evaporation residues (%)	61	≥60
Penetration (100 g, 25 °C, 5 s) (0.1 mm)	75	40–100
Softening point (°C)	60	≥53
5 °C ductility (cm)	32	≥20
Solubility (%)	99.5	≥97.5
1 d storage stability (%)	0.1	≤1
5 d storage stability (%)	1.2	≤5

**Table 4:** Basic properties of aggregates.

	Particle Size/mm	10–15	5–10	3–5	0–3
Indicators					
Apparent density (g·cm^−3^)		2.678	2.680	2.677	2.706
Bulk density (g·cm^−3^)		2.625	2.607	2.595	-
Dry apparent density (g·cm^−3^)		2.645	2.635	2.625	-
Flat and elongated particle content (%)		6.1	4.4	-	-
Water absorption (%)		0.75	1.04	1.19	-

**Table 5:** Basic technical indicators of mineral powder.

Indicators		Test Values	Technical Requirements
Bulk specific gravity (g·cm^−3^)		2.798	≥2.5
Moisture content (%)		0.82	≤1
Particle size range (%)	<0.6 mm	100	100
	<0.15 mm	93.6	90–100
	<0.075 mm	80.2	75–100

**Table 6:** Gradation used in the test.

Sieve Size (mm)	16	13.2	9.5	4.75	2.36	1.18	0.6	0.3	0.15	0.075
Passing rate (%)	100	96.2	76.3	48.8	31.3	21.0	15.7	11.4	9.5	6.1

## Data Availability

The raw and processed data required to reproduce these results are available by contacting the authors.
